# Application of bilateral tDCS over left and right M1 produces asymmetric training and retention effects when learning a rhythmic bimanual task

**DOI:** 10.1007/s00221-025-07045-4

**Published:** 2025-03-14

**Authors:** Austin T. McCulloch, David L. Wright, John J. Buchanan

**Affiliations:** 1 Department of Kinesiology and Sport Management Perception-action Dynamics Laboratory, Texas A&M University, College Station, TX 77843 USA; 2 Department of Health and Sport Studies McPherson, McPherson College, McPherson, KS 67460 USA; 3 Department of Kinesiology and Sport Management, Texas A&M University, College Station, TX 77843 USA

**Keywords:** Cortical excitability, Consolidation, Relative phase, Movement amplitude

## Abstract

Many motor skills require precise coordination between the arms to accomplish. The use of transcranial direct current stimulation (tDCS) has helped to reveal hemispheric contributions to bimanual skills. In this study, three bilateral montages were used to explore hemispheric contributions to a rhythmic bimanual skill: anode left M1/cathode right M1 (LARC), anode right M1/cathode left M1 (RALC), and sham. Stimulation lasted 20-minutes during training. Retention was examined 6-hr after training. Participants (*n* = 46) learned a bimanual 90° relative-phase pattern with a half-cycle movement amplitude goal of 12 cm per arm at self-selected movement frequencies. Greater coordination variability in the 90° pattern emerged early under RALC compared to LARC, with no difference in performance accuracy. Larger movement amplitudes emerged in training with LARC compared to sham but not compared to RALC. tDCS montage had no impact on coordination variability and accuracy of the 90° pattern after the 6-hr delay. Montage was associated with a delayed movement amplitude effect emerging in retention, with larger amplitudes in LARC compared to RALC and sham. The asymmetries observed across training and retention emerged from of an interaction between tDCS and the left-hemisphere’s role in the control of bimanual movements in right-handed individuals.

**Supplementary Information:**

The online version contains supplementary material available at 10.1007/s00221-025-07045-4.

## Introduction

Bimanual actions are a ubiquitous component of our motor skill set that allow us to manipulate and interact with objects in the world, from buttoning a shirt, to drying dishes or playing the drums. Precision and accuracy of bimanual actions requires the control of movement time, force, spatiotemporal coordination between the joints/limbs, and movement amplitude. As a skill set, research indicates that bimanual actions are not simply the linking together of two independent unimanual actions (Kelso et al. 1979a, b; Sherwood and Nishimura 1999; Ryu and Buchanan 2004). The combined movement of both arms has been shown to influence the stability of spatiotemporal coordination patterns and the production of movement amplitudes and thereby influence successful task completion (Buchanan and Ryu 2005, 2012; de Poel et al. 2020). Research has shown that intercortical and intracortical neural activation patterns may underlie the across limb influences seen when the two arms work together (Ivry et al. 2004; Swinnen and Wenderoth 2004; Heitger et al. 2012; Rueda-Delgado et al. 2014). Transcranial direct current stimulation (tDCS) affects underlying cortical network excitability (Nitsche and Paulus 2000b, 2001). The current study applied tDCS bilaterally over the hand areas of the primary motor cortex (C3 and C4 of the 10–20 EEG system) to explore whether this technique would influence emerging performance and consolidation of a rhythmic bimanual task. The specific behavioral variables examined were bimanual relative phase (φ, a measure of spatiotemporal coordination) and bimanual movement amplitude.

tDCS uses low voltage electrical currents developed with a positive (anode) and a negative (cathode) electrode to stimulate underlying brain tissue. The applied current flows from the anode to cathode and induces intracerebral current flow, allowing the target brain region to be modified in a polarity specific manner (Nitsche and Paulus 2000a). Stimulation intensity is measured in milliampere (mA) and is typically applied from several minutes and up to 20 min per exposure. Anodal stimulation has been demonstrated to increase neural excitability whereas cathodal stimulation inhibits excitability (Nitsche and Paulus 2000a, 2001). When administering tDCS an important decision is when to apply stimulation, before, during or after training. In the case of unimanual tasks, tDCS has been applied to M1 after and during training leading to enhanced rate and strength of consolidation in serial reaction time tasks (Tecchio et al. 2010; Kim and Wright 2020) and force controlled aiming tasks (Reis et al. 2009). Research with bimanual learning tasks have used either stimulation before or during training.

A common protocol using tDCS before-training is to first conduct a behavioral pre-test, followed by stimulation with no movement, then proceed with training, and follow up with a behavioral post-test scenario where the difference in pre- and post-test performance is used as a measure of the influence of tDCS on neuromodulation over the targeted area (Furuya et al. 2013, 2014; Gomes-Osman and Field-Fote 2013; Carter et al. 2015, 2017; McCambridge et al. 2016). The before training stimulation protocol has produced mixed results, from no performance effects in bimanual serial timing tasks (Furuya et al. 2013; McCambridge et al. 2016), enhanced performance in synchronous bimanual force matching tasks (Jin et al. 2019b), enhanced performance in controls but not in expert pianists in serial bimanual tasks (Furuya et al. 2014), and finally enhanced performance in non-learning contexts using in-phase and anti-phase bimanual patterns (Carter et al. 2015, 2017). The Carter et al. studies have relevance here in that they showed increased stability for a rhythmic anti-phase (asynchronous index finger flex-extend motions, φ = 180°) bimanual pattern but not an in-phase bimanual pattern (synchronous flex-extend motions, φ = 0°) after tDCS was applied to SMA. Both the in-phase and anti-phase patterns may be viewed as intrinsically stable, with in-phase a more stable pattern than anti-phase (Haken et al. 1985; Kovacs et al. 2009b) leaving little room for improvement in the in-phase pattern. The bimanual pattern used in the present study was a 90° relative phase pattern that can require extensive training to perform in a stable manner (Schöner et al. 1992; Zanone and Kelso 1992) except under specific visually based feedback conditions (Kovacs et al. 2009a; Kovacs and Shea 2011).

Applying tDCS during bimanual training has also produced mixed results. In a study using the Purdue Pegboard task, application of bi-hemispheric tDCS (dual-anodal left and right M1, with 6 cathode positions) during training improved bimanual performance when the hands moved synchronously but not when the hands moved asynchronously (Pixa et al. 2017b). In a cup stacking bimanual task with 3 training days, bi-hemispheric tDCS (dual-anodal left and right M1) applied during training improved performance on only one of two stacking tasks (Pixa et al. 2017a). In force matching tasks, consistency in performance improvements with the use of tDCS have been found. For example, left M1 anodal stimulation was found to increase both force maintenance accuracy and rhythmic alteration of force for discrete and rhythmic tasks when pressing a rigid bar synchronously with both hands (Jin et al. 2019a). In synchronous bimanual grip force tasks, left M1 anodal stimulation improved force matching and helped to coordinate motor force synergies (Lee and Kang 2023). Most recently, left M1 anodal stimulation was associated with a reduced movement time in a synchronized bimanual target aiming task (Rizvi et al. 2023), and a reduction in errors without an increase in speed in a bimanual serial typing task (Sevilla-Sanchez et al. 2021). However, multi day application of anodal tDCS to left M1 when training on rhythmic bimanual multifrequency patterns of 1:1, 2:1 and 3:1, frequency patterns resulted in no performance advantage compared to sham (Vancleef et al. 2016). For the 1:1 ratio, the bimanual patterns were in-phase and anti-phase, and as noted earlier both patterns are intrinsic, with in-phase more stable than anti-phase. It was found that tDCS over the SMA only influenced anti-phase stability (Carter et al. 2015, 2017). As for the 2:1 and 3:1 multifrequency patterns, the use of a montage consisting of left M1 anode and right supraorbital cathode might have been an important design feature that differed from bimanual studies that used left M1 anode and right M1 cathode and reported positive tDCS results with synchronous bimanual force control tasks (Hikosaka and Aramaki 2021; Lee and Kang 2023).

The work reviewed suggests that the application of tDCS during training may provide the best opportunity to see performance changes as a bimanual task is acquired, with possible concomitant performance benefits following a period of consolidation. Motor learning studies (unimanual and bimanual) have demonstrated consolidation of trained skill level following a 4-to-6-hour window from the end of training until a retest (Brashers-Krug et al. 1996; Kim et al. 2021b; McCulloch et al. 2021). Work in the bimanual domain has not identified a specific tDCS montage that is more advantages to producing short- or long-term changes. Recent work showing an effect on synchronous bimanual force output used a bilateral montage with the anode over left M1 and the cathode over right M1 (LARC) (Hikosaka and Aramaki 2021; Lee and Kang 2023). The montages used in the present work were the LARC, and a montage with the cathode over left M1 and anode over right M1 (RALC). tDCS was applied during training, and performance was evaluated during training and after a 6-hr consolidation period. The bimanual task consisted of a 90° relative phase (spatiotemporal) goal and a half-cycle amplitude goal of 12 cm. The 90° relative phase pattern is not characterized by a ceiling effect like that observed for in-phase and anti-phase patterns used in previous studies applying tDCS (Carter et al. 2015, 2017; Vancleef et al. 2016).

Neurophysiological evidence (fMRI, EEG) demonstrates that the left-hemisphere plays a significant role in coupling bimanual actions (Serrien et al. 2003, 2006). The main hypothesis is that the LARC montage will have more of an influence on bimanual performance over the RALC and sham montages. The key issue is whether the performance influence occurs in the spatiotemporal aspect of coordination or movement amplitude. The Vancleef et al. (2016) findings suggest the spatiotemporal component may not benefit from tDCS, however, the use of the more difficult 90° pattern compared to in-phase and anti-phase may allow for performance changes to occur. Specifically, it is hypothesized that the LARC montage will be associated with more accurate and stable performance of the 90° spatiotemporal pattern compared to the RALC and sham montages during training. The possible influence of tDCS on learning a required movement amplitude has not been explored. Research has shown that movement amplitude in rhythmic tasks is controlled independently of relative phase (Ryu and Buchanan 2004; Buchanan et al. 2007; Pan and Van Gemmert 2019). This suggests the possibility that tDCS may influence movement amplitude during training. Research has shown that tDCS increases the excitability in the area under anode stimulation (Nitsche and Paulus 2000a). It is hypothesized that the LARC montage will result in larger movement amplitudes compared to the RALC and sham montages. Any changes associated with the LARC are predicted to still be observable in the retest based on work showing that tDCS of M1 influences off-line consolidation in other motor skill tasks (Robertson et al. 2004; Reis et al. 2009; Robertson 2009; Kim et al. 2021b).

## Materials and methods

### Participants

A total of 46 participants that passed the initial screening test for application of tDCS were recruited over a 13-week interval in a standard 15-week semester. Participants were young adults (mean age = 21 yrs, with a std. dev. of 1.7 yrs.; female *n* = 32) and free of neuromuscular disorders which inhibit upper limb movement or sensation. The participants were right hand dominant with a mean laterality quotient score of 92.9 (std. dev. = 9.49) (Oldfield 1971; Coren 1993). The experimental procedure, consent form, and all questionnaires were approved by the Human Subjects Internal Review Board at Texas A&M University. All participants voluntarily consented in line with the Helsinki Declaration and received class credit for kinesiology courses.

### Procedures

**Fig. 1:**
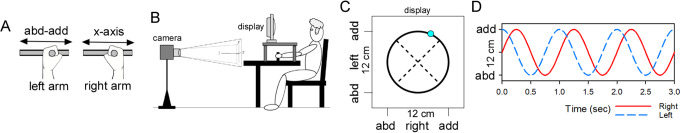
**A-D. (A)** Orientation of the arms, handles, and movement direction are represented. Abduction-adduction motion of the handles on the horizontal plane (*x-axis*) is defined with respect to the body midline and required motion of the whole arm. **(B)** The positioning of a participant with respect to the camera, feedback display, and handles is Portrayed. **(C)** The Lissajous plot feedback display. The crossed lines represent in-phase (positive slope) and anti-phase (negative slope) coordination for the familiarization trials, and the circle represents a 90° relative phase for the training and retest trials. **(D)** The two simulated time series representing right, and left arm motion have a 90° phase offset with an amplitude of 12 cm, which would move the dot around the circle in (**C**)

*Task and data collection.* Sitting in an upright and comfortable position, participants grasped two vertical handles and slid them along a fixed track mounted on a desk (Fig. 1A). The handles were constrained to move laterally on the horizontal plane with abduction-adduction motion of the handles defined with respect to the body midline. An Optotrak Certus 3D camera system (Northern Digital, Inc.) was used to record the motion of two infra-red LEDs attached to the vertical handles. The camera was positioned approximately two meters from the participants’ hands and at a height of approximately one meter with the *z* and *x*-axes parallel to the floor (Fig. 1B). Augmented visual feedback was displayed via a Lissajous plot on a computer monitor placed directly in front of the participant (approximately 60 cm) at eye-level. The horizontal displacement of the right (RA) and left (LA) arms was mapped to the *x*-axis and *y*-axis of the Lissajous plot, respectively (Fig. 1C). Six familiarization trials were performed, consisting of three in-phase trials (RA and LA abduct and adduct together) and three anti-phase trials (RA abducts as the LA adducts) with a visual guide of a 45° positive or negative sloped line, respectively (Fig. 1C). The bimanual training pattern was a 90° relative phase pattern defined with the circle template in the Lissajous plot, requiring participants to create a ¼ cycle lag between the two arms (Fig. 1D). The 1/4 cycle lag was not preset before the trial, with all participants starting from a neutral position of the handles. Vision of the arms was blocked.

Participants received verbal instructions and were provided concurrent visual feedback in the form of a dot moving in the Lissajous plot. The learning required in this experiment may be classified as discovery. The instructions given to the participants emphasized using the Lissajous plot as the guide to achieve the correct coordination pattern by moving the dot along the template, and not inside, outside, or beside the template. Movement of the dot along the template would meet the required spatiotemporal goal of a 90° relative phase and a half-cycle movement amplitude goal of 12 cm. Neither of the numerical goals were given to the participants, a feature consistent with previous work using this display (Kovacs et al. 2009a; McCulloch et al. 2021). The task required full arm motions to accomplish the task, with primary motion about the elbow and shoulders to generate the lateral handle motion (a complete cycle 24 cm) to move the dot on the circle template (Fig. 1B and C). Movement frequency was self-selected, and participants were instructed to increase frequency over training.

The LEDs attached to the vertical handles were sampled at 100 Hz. All dependent measures were calculated using Matlab. The *x*-axis time series of the markers were dual pass filtered (Butterworth). Performance was evaluated by calculating the continuous relative phase between LA and RA motions based on the *x*-axis time series. The *x*-axis time series for each arm (*x*_*i*_) was differentiated to produce a velocity signal ($\:{dx}_{i}/{dt}_{i}$). The displacement and velocity time series were normalized to the range − 1, 1 on a half-cycle basis (Varlet and Richardson 2011) before computing the individual phase angles for the left (*θ*_*l*_) and right (*θ*_*r*_) arms: $\:{\theta\:}_{i}\:=\:{tan}^{-1}\:\left[{dx}_{i}/\left({dx}_{i}/{dt}_{i}\right)\right]$ (Scholz and Kelso 1989). To evaluate performance improvements in the task, continuous relative phase ($\:{\varphi\:}_{c}$) was calculated by subtracting the left arm phase angle from the right arm phase angle, $\:{\varphi\:}_{c}=\:{\theta\:}_{r}-\:{\theta\:}_{l}$. The $\:{\varphi\:}_{c}$ time series was submitted to a circular transformation and two performance measures were calculated from the $\:{\varphi\:}_{c}$ time series: (1) performance accuracy was evaluated with a time-on-task bandwidth computed as the percentage of time in a trial that the $\:{\varphi\:}_{c}$ time series fell between 112.5° and 67.5° (±22.5° around the 90° training pattern, or BW_22_) (Wilson et al. 2010; McCulloch et al. 2021); and (2) a coordination variability (stability) measure defined as the standard deviation of mean relative phase $\:\left({\varphi\:}_{SD}\right)$ computed from the complete $\:{\varphi\:}_{c}$ time series. The bandwidth measure represents spatiotemporal performance accuracy, and the standard deviation represents spatiotemporal (coordination) stability. Movement frequency was determined with a discrete Fast Fourier Transform (FFT) of the *x*-axis time series for the right and left arms’ motion. The frequency of the largest peak returned from the FFT was taken to represent the average cycling frequency for each arm in a trial. The frequency values were averaged for the two arms by trial and the analysis of the averaged frequency (FRQ) data is reported in the results. The two *x-axis* time series were mean centered and detrended and plotted as a Lissajous plot. For each pair of points, *LAx*_*i*_ and *RAx*_*i*_, the radius from the origin of (0,0) was computed as the measure of bimanual movement amplitude. The individual radii from a trial were averaged to create a mean movement amplitude (AMP) with a target of 6 cm. Lateral movement amplitude was computed from the two *x-axis* times series from the two handles thereby representing arm motion. The results focus on the bimanual measures, FRQ and AMP. For analysis of individual arm motion see supplemental data (sections S1 and S2).

**Fig. 2:**
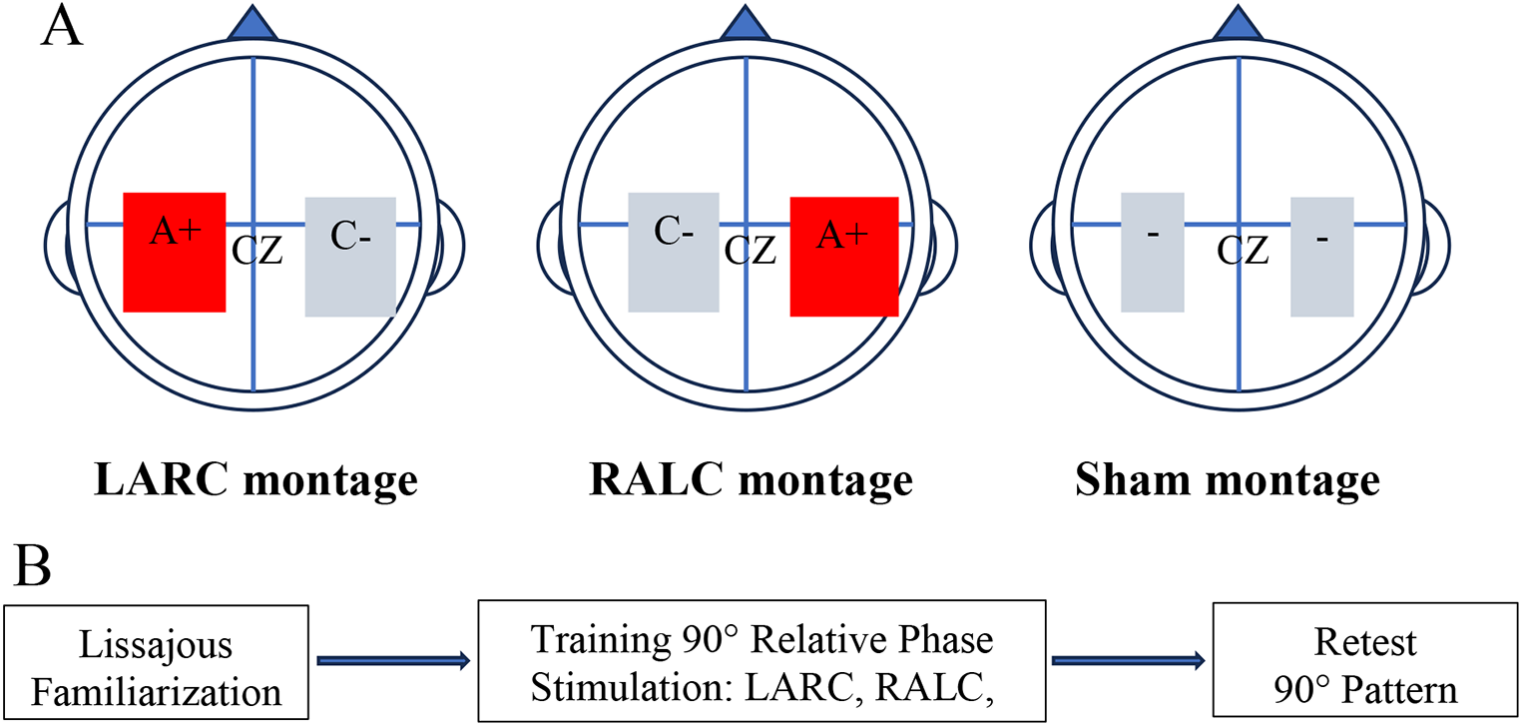
**A-B**. **A)** The three tDCS montages used in this experiment are depicted: (1) LARC– left M1 anode (A+) and right M1 cathode (**C**-), (2) RALC right M1 anode (A+) and left M1 cathode (**C**-), and (3) sham. The direction of current flow is from the red to blue square for the LARC and RALC montages. The sham condition montage is shown with two squares with minus signs to indicate no prolonged stimulation (only ramp up– ramp down current) during the training period. **B)** The flow chart represents the experimental timeline, from familiarization trials (in-phase and anti-phase) to stimulation and training of 90° to retest of 90° after a 6-hr consolidation window

*Transcranial direct current stimulation*. A 1 × 1 low-intensity transcranial electrical stimulator system (Soterix Medical, New York, NY) was used to deliver direct current stimulation to modulate cortical excitability during training. The 2-mA current (Furuya et al. 2014; Jin et al. 2019a) was transferred by anodal and cathodal conductive rubber electrodes (5 × 5 cm) resulting in a maximum current density of 0.08 mA/cm. The electrodes were placed in sponges soaked in 5 mL of a 0.9% saline solution. The anode/cathode electrodes were centered over target areas C3 (left hemisphere) and C4 (right-hemisphere) of the 10–20 EEG system (Fig. 2A): (1) LARC group, C3 anode and C4 cathode (*n* = 16), and (2) RALC group, C3 cathode and C4 anode (*n* = 16) (Furuya et al. 2013, 2014; McCambridge et al. 2016). As modeled by the HD-Explore software (Soterix Medical Inc.), M1 under the anode (C3 or C4) should have heightened field intensity resulting from the LARC and RALC montages. The sham condition was pseudo randomly chosen as one of these two combinations (*n* = 14). The electrodes were placed at a minimal distance of 6 cm to decrease the probability of shunting current through the scalp (Rush and Driscoll 1968). In the LARC and RALC conditions, stimulation was for 21 min with a ramping up (start) and down (end) over 30-second intervals. In the sham condition, current ramped up (start) for 30 s to reach 2 mA and then ramped down (end) for 30 s, with no stimulation over the intervening 20-minute training period.

Application of tDCS was single blind in this experiment (Lowenthal-Raz et al. 2024; Yang et al. 2024). All participants (RALC, LARC, and sham) were told they may feel itching, tingling, warmth, etc. associated with the application of tDCS throughout training. A post training survey of the effects of tDCS was undertaken and each participant asked if they experienced real or sham tDCS as part of the survey (Vancleef et al. 2016) (see supplemental information section S3).

*Training and retention*. This experiment investigated the training (20-min) and consolidation (6-hr retest interval) processes underlying the development of a motor memory for a bimanual 90° relative phase pattern with a required half-cycle amplitude of 12 cm. The experiment required participants to partake in two separate sessions separated by 6 hours (Fig. 2B). Session 1, the training session, lasted between 60 and 80 min while session 2, the retest session, lasted between 20 and 30 min. Every training-retest trial lasted 20 s and was followed by a 20 s rest. Before the training session, participants completed six familiarization trials with the Lissajous plot providing concurrent feedback: three in-phase trials ($\:\varphi\:$=0°), three antiphase trials ($\:\varphi\:$=180°). After the familiarization trials, the tDCS electrodes were secured to the participant’s scalp. Stimulation (active and sham) was applied during the 21 training trials (T1-T21). The Lissajous plot/cursor provided on-line feedback during training. After a 6-hr delay, participants returned to the lab to complete four retest trials (R1-R4) performed with the Lissajous plot/cursor feedback display, but without applying tDCS. The retest trials provide an estimate of the strength of the motor memory for the trained 90° relative phase pattern and required 12 cm arm motions.

### Statistical analyses

Every three training trials starting with the first and ending with the 21st were averaged to create seven training blocks (B1-B7) for each performance measure (BW_22_, $\:{\varphi\:}_{SD}$, AMP, and FRQ) and the block means were submitted to statistical analysis. To evaluate if M1 stimulation influenced training performance, the blocked behavioral measures were analyzed in separate repeated measures ANOVAs with Stimulation Montage as a between factor (LARC, RALC, sham) and training Block as a repeated within factor (B1-B7). To determine if M1 stimulation influenced post-training retention performance, the four retention trials were averaged and the performance measures (BW_22_, $\:{\varphi\:}_{SD}$, AMP, and FRQ) were analyzed with one-way ANOVAs with Stimulation Montage as the factor. To examine if the performance level changed over the retention interval, a delta score (Δ) was created for each measure. The last four training trials (P_avg_) and the four retention trials (R_avg_) were averaged for each subject and a Δ value was computed as, Δ = P_avg_ - R_avg_, for each performance measure. The Δ values were analyzed with one-way ANOVAs with Stimulation Montage as the factor to determine if off-line stabilization or off-line enhancement emerged.

All repeated measures ANOVAs (procedure mixed) and one-way ANOVAs (procedure GLM) were performed with SAS statistical software (9.4). For the repeated ANOVAs, Sphericity was checked with Mauchly’s test, and all repeated ANOVAs performed violated the sphericity assumption, as a result all *p* values reported are Greenhous-Geiser adjusted. Significant main effects and interactions were explored with the least squares mean method using the Tukey-Kramer adjustment as a post-hoc test procedure with an acceptance level of *α = 0.05*. For the one-way ANOVAs, homogeneity of variance was tested with Leven’s test, and it was not met in any of the ANOVAs, therefore *F* and *p* values reported are from Welch’s test. Student-Newman-Keuls test (*α = 0.05*) was used as the post-hoc test when appropriate for the one-way ANOVAs. The effect size of all results was estimated with partial eta-square (*η*^*2*^_*p*_) as follows: *η*^*2*^_*p*_ > 0.01 small effect, *η*^*2*^_*p*_ > 0.06 medium effect, and *η*^*2*^_*p*_ > 0.14 large effect (Cohen 1998).

### Results

### Training performance

**Fig. 3:**
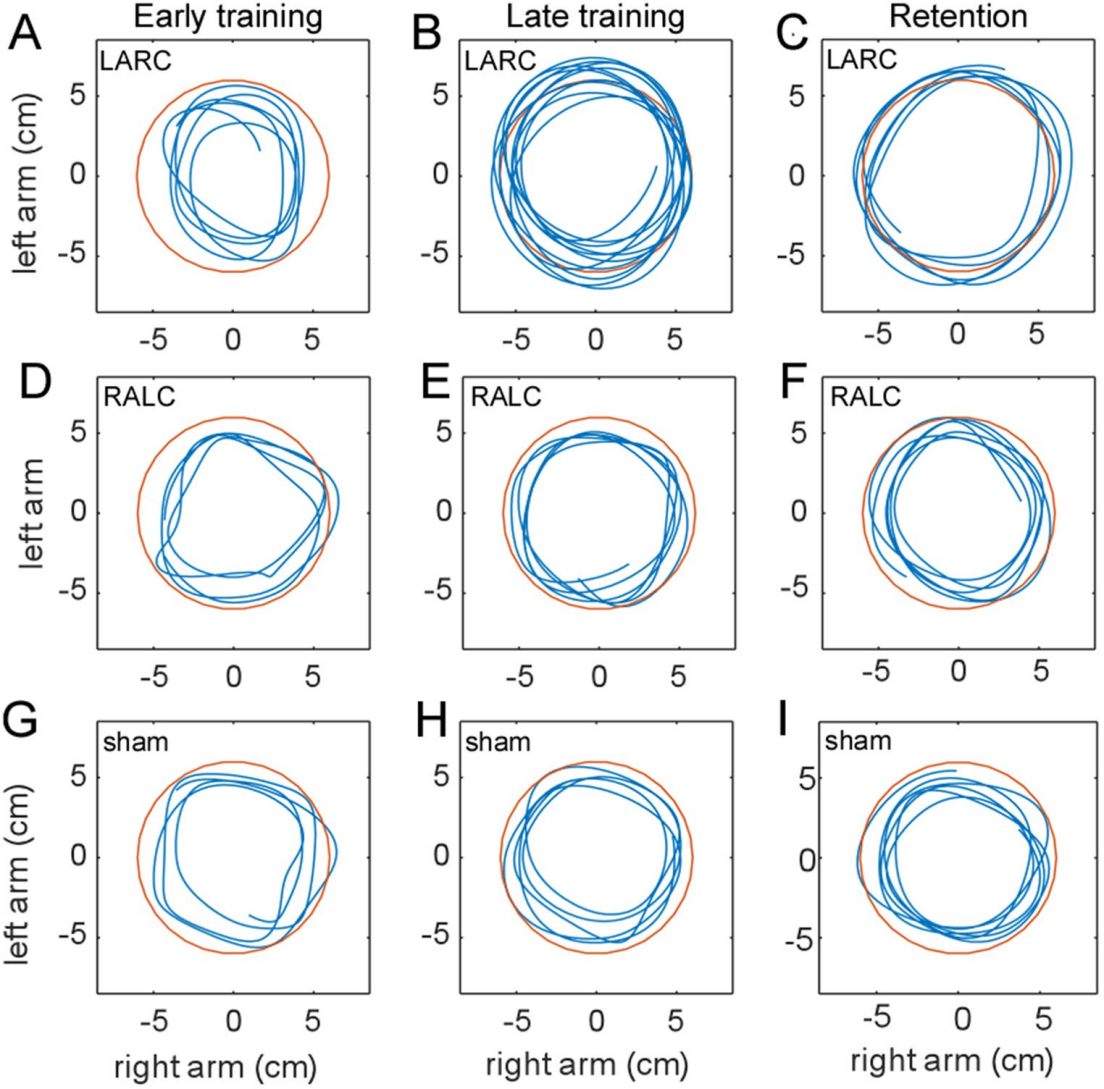
**A-F.** Sample trajectories from an individual in the LARC group (**A**, **B**, **C**), RALC group (**D**, **E**, **F**), and sham group (**G**, **H**, **I**). The trials in **A** (trial 4), **D** (trial 7), and **G** (trial 6) are from early in training, and the trials in **B** (trial 21), **E** (trial 21), and **H** (trial 14) are from later in training. The trials in **C** (trial 4), **F** (trial 2) and **I** (trial 4) are examples from the four retest trials, 6-hrs. after the end of practice. Left-arm motion is plotted on the y-axis and right-arm motion on the x-axis. Half-cycle displacement was 12 cm

As expected, the three groups were characterized by significant performance improvements of the 90° relative phase pattern and required 12 cm amplitude over the training interval, with self-scaled movement frequency increasing (Fig. 3). The issue at hand is the influence of applying tDCS during training on the rate and extent of performance improvements.

**Fig. 4:**
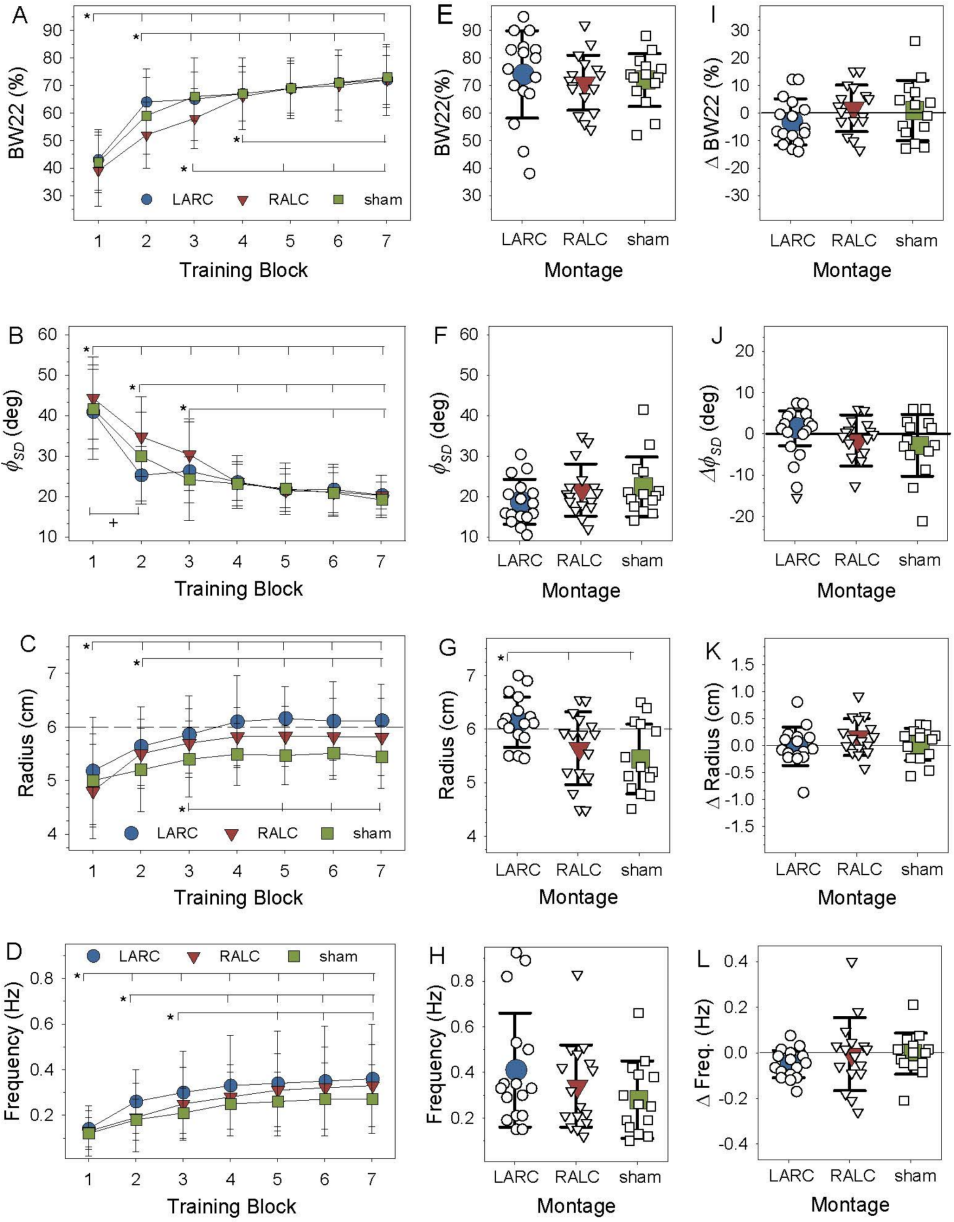
**A-L.** In all plots, LARC data is plotted as a circle, RALC as a triangle, sham as a square. **A-D)** Training data plotted by Block and Montage. **E-H**) Retention data plotted by Montage, group means and individual participant data. **I-L**) Delta values representing performance difference between the last 4 training trials and the 4 retention trials, group means, and individual participant data are plotted. **A**, **E**, and **I**) BW22 - relative phase accuracy measure. **B**, **F**, and **J)**$\:{\varphi\:}_{SD}$ - Coordination variability measure. **C**, **G**, and **K**) AMP - bimanual amplitude measure (radius). **D**, **H**, and **L**) FRQ– averaged frequency across both arms. The error bars represent standard deviation of the group mean. The asterisks and brackets represent significant differences between blocks in **A**, **C** and **D**. In **B**, within group differences found in the interaction tests are highlighted for the RALC (*) and LARC (+) montages

*Spatiotemporal coordination: Relative phase.* Training improved performance accuracy in all three groups. The ANOVA performed on the bandwidth measure (BW_22_) around the target relative phase of 90° found a significant main effect of Block, *F (6*,* 258)* = *96.22*, *p* <.*0001*, $\:{n}_{p}^{2}=$*.69* (Fig. 4A). Post-hoc *t-*values (Tukey-Kramer) are significant with adjusted *p* <.0001 unless specifically reported. Significant differences in performance accuracy occurred between blocks: *B1* < *B2 (t (258) = -10.8)*, B1 < B3 (*t = -13.9*), B1 < B4 (*t = -16.4*), B1 < B5 (*t = -17.9)*, B1 < B6 (*t = -18.9*), B1 < B7 (*t = -20.1*), B2 < B3 *(t = -3.1*, *p=.04*), B2 < B4 (*t = -5.4)*, B2 < B5 (*t = -7.1)*, B2 < B6 (*t = -8.0)*, B2 < B7 (*t = -9.2)*, B3 < B5 *(t = -4.02*, *p* =.*0015*), B3 < B6 (*t = -4.9)*, B3 < B7 (*t = -6.1)*, and B4 < B7 (*t = -3.6*, *p* =.*0054*). Post-hoc tests did not reveal significant differences in performance accuracy when comparing block B4 to B5 and B6, or when comparing between training blocks B5, B6, and B7. The main effect of Montage was non-significant (*F (2*,* 28) = 0.45*,* p =.63*, $\:{n}_{p}^{2}=$*0.03 )*, and the Montage × Block interaction approached a standard level of significance (*F (12*,* 258) = 1.75*, *p =.057*, $\:{n}_{p}^{2}=$*0.08).*

With training, bimanual coordination at 90° became more stable in all three groups. The ANOVA performed on $\:{\varphi\:}_{SD}$ the coordination variability measure found a significant main effect of Block, *F (6*,* 258) = 84.51*, *p <.0001*, $\:{n}_{p}^{2}=$*0.66*. The Montage × Block interaction was significant, *F (12*,* 258) = 1.95*, *p =.029*, $\:{n}_{p}^{2}=$*0.09* (Fig. 4B). Post-hoc *t-tests* (Tukey Kramer) were used to examine the interaction and *t-*values shown were significant with adjusted *p* <.0001 unless specifically reported. This interaction was examined by comparing across blocks within a montage and each montage had a distinct drop off in variability across blocks: (1) LARC, B1 > B2 (*t (258) = 7.69*), B1 > B3 (*t = 7.18*), B1 > B4 (*t = 8.5*), B1 > B5 (*t = 9.45*), B1 > B6 (*t = 9.41*), and B1 > B7 (*t = 10.10*), with no significant differences between blocks 2 through 7; (2) RALC B1 > B2 (*t = 4.69*, *p=.0008*), B1 > B3 (*t = 6.89*), B1 > B4 (*t = 10.2*), B1 > B5 (*t = 11.25*), B1 > B6 (*t = 11.41*), B1 > B7 (*t = 11.83*), B2 > B4 (*t = 5.54*), B2 > B5 (*t = 6.56*), B2 > B6 (*t = 6.72*), B2 > B7 (*t = 7.14*), B3 > B5 (*t = 4.37*, *p=.003*), B3 > B6 (*t = 4.52*, *p =.002*), and B3 > B7 (*t = 4.94*, *p =.0003*), with no significant differences when testing comparisons across blocks 4 through 7; and (3) sham B1 > B2 (*t = 5.39*), B1 > B3 (*t = 8.1*), B1 > B4 (*t = 8.5*), B1 > B5 (*t = 9.1*), B1 > B6 (*t = 9.55*), B1 > B7 (*t = 10.34*), B2 > B5 (*t = 3.68*, *p =.04*), B2 > B6 (*t = 4.1*, *p =.008*), and B7 (*t = 4.95*, *p =.0003*), with no significant differences when testing across blocks 3 through 7. This interaction was also examined comparing the 3 montages at each block, with the only significant difference in B2 with RALC > LARC (*t (258) = -3.69*, *p =.038*). The main effect of Montage was non-significant (*F (2*,* 28) = 0.95*, *p =.84*, $\:{n}_{p}^{2}=$*0.07)*.

*Movement amplitude*. Bimanual movement amplitude (AMP) increased in the three groups with practice. The ANOVA performed on the AMP data found significant main effects of Montage, *F (2*,* 28) = 3.93*, *p =.031*, $\:{n}_{p}^{2}=\:$*0.21*, and Block, *F (6*,* 258) = 21.69*, *p <.0001*, $\:{n}_{p}^{2}=$*.34* (Fig. 4C). Post-hoc tests of the Montage effect revealed significantly larger amplitude in the LARC group compared to sham (*t(28)=2.75*, *p=.027*), with no difference between RALC and LARC (*p*=.*43*) or RALC and sham (*p=.37*). The block effect was examined with post-hoc *t-*tests (Tukey-Kramer) and *t* values shown are significant with adjusted *p* <.0001 unless specifically reported. The following significant differences across blocks were found: B1 < B2 (*t (258) = -5.3*), B1 < B3 (*t = -7.4*), B1 < B4 (*t = -8.7*), B1 < B5 (*t = -8.9*), B1 < B6 (*t = -8.8*), B1 < B7 (*t = -8.6*), B2 < B4 (*t =-3.4*, *p =.011*), B5 (*t =-3.6*, *p =.006*), B6 (*t = -3.58*, *p =.007*), and B7 (*t = -3.31*, *p =.018*), with no significant differences found across blocks 3, 4, 5, 6, and 7. The Montage × Trial interaction was non-significant (*F(12*,* 258) = 1.34*, *p =.19*, $\:{n}_{p}^{2}=$*0.06*).

*Movement frequency.* Self-paced bimanual movement frequency (FRQ) increased significantly with practice across the three groups. The ANOVA performed on the FRQ data found a significant main effect of Block, *F (6*,* 258) = 38.21*, *p <.0001*, $\:{n}_{p}^{2}=$*.47* (Fig. 4D). The block effect was examined with post-hoc *t-tests* (Tukey-Kramer) and *t* values shown are significant with adjusted *p* <.0001 unless specifically reported. The following significant differences between blocks were found, B1 < B2 (*t (258) = -5.1*), B1 < B3 (*t = -7.6*), B1 < B4 (*t = -9.8*), B1 < B5 (*t = -11.0*), B1 < B6 (*t = -11.70*), B1 < B7 (*t = -12.07*), B2 < B4 (*t = -4.68*), B2 < B5 (*t = -5.88*), B2 < B6 (*t = -6.57*), B2 < B7 (*t = -6.94*), B3 < B5 (*t = -3.34*, *p =.016*), B3 < B6 (*t = -4.02*, *p =.0015*), and B3 < B7 (*t = -4.39*, *p =.0003*), with no significant differences found between blocks 4, 5, 6, and 7. The main effect of Montage (*F (2*,* 28) = 0.48*, *p =.62*, $\:{n}_{p}^{2}=$*0.03)*, and the Montage × Block interaction (*F(12*,* 258) = 0.52*, *p =.89*, $\:{n}_{p}^{2}=$*0.02*) were non-significant.

### Retention performance

*Spatiotemporal coordination: Relative phase.* The analysis of the retention data revealed results consistent with training for the two spatiotemporal performance measures. Regarding performance of the 90° bimanual pattern, the ANOVA of the performance accuracy measure (BW_22_) revealed no significant differences as a function of Montage, *F (2*,* 28.1) = 0.22*, *p =.80*), and the ANOVA of the stability measure ($\:{\varphi\:}_{SD}$) revealed no significant differences as a function of Montage, *F (2*,* 27.5) = 1.52*, *p <.23*, $\:{n}_{p}^{2}=$*.06* (Fig. 4E, 4F).

*Movement amplitude and frequency.* The analysis of the retention data revealed a 6-hr. delay effect of montage stimulation in the AMP data with no delay effect in the FRQ data. The ANOVA performed on the bimanual AMP data found a significant main effect of Montage, *F (2*,* 27.1) = 6.05*, *p =.0067*, $\:{n}_{p}^{2}=\:$*.19* (Fig. 4G). The post-hoc tests of the Montage effect (*p* <.*05*) revealed that bimanual movement amplitude in the LARC group was significantly larger compared to the RALC and sham groups. There was no significant difference between RALC and sham. The ANOVA of the FRQ data did not reveal a significant effect of Montage, *F (2*,* 28.3) = 1.32*, *p =.26*, $\:{n}_{p}^{2}=\:$*.06* (Fig. 4H)

*Delta: performance change*. The analysis of the Δ values revealed no significant change in performance for the spatiotemporal variable relative phase, with no significant differences in accuracy (BW_22_), *F (2*,* 28.3) = 1.32*, *p =.26*, $\:{n}_{p}^{2}=\:$*0.06*, or stability ($\:{\varphi\:}_{SD}$), *F (2*,* 28.3) = 1.32*, *p =.26*, $\:{n}_{p}^{2}=\:$*0.06*, associated with Montage (Fig. 4I, 4J). The analysis of the bimanual AMP data, *F (2*,* 28.3) = 1.32*, *p =.26*, $\:{n}_{p}^{2}=\:$*0.06*, and FRQ data, *F (2*,* 28.3) = 1.32*, *p =.26*, $\:{n}_{p}^{2}=\:$*0.06*, also revealed no significant effect linked to Montage (Fig. 4K, 4L).

## Discussion

The results from the current study revealed hemispheric asymmetries in bimanual performance associated with bilateral placement of anode and cathode electrodes over left and right M1 (C3 and C4) during motor skill training. The hypothesis that the LARC montage would produce different performance outcomes compared to RALC and sham was supported to an extent. The specific hypothesis about spatiotemporal coordination was only partly supported by the differences that emerged in the coordination variability ($\:{\varphi\:}_{SD}$) measure during early training. No end of training or retest effects emerged in the spatiotemporal measures. The hypothesis about movement amplitude was supported in that larger bimanual radius (AMP) amplitudes and larger individual arm abduction-adduction amplitude measures emerged under LARC in training. The amplitude differences were still present in the retest data. The spatiotemporal and movement amplitude components of the task were the trained variables. The use of tDCS did not influence the increase in self-paced movement frequency that occurred across practice. Participants were right-hand dominant, thus the differences uncovered may point to laterality effects associated with the left-hemisphere as dominant and the right-hemisphere as non-dominant (Serrien et al. 2003, 2006; Schambra et al. 2011; King et al. 2020).

### Training performance

Spatiotemporal coordination was measured through the analysis of the relative phase relationship between the two arms. The targeted relative phase was a 90° pattern that is not intrinsically stable like in-phase and anti-phase bimanual coordination (Zanone and Kelso 1992). To facilitate rapid training a Lissajous plot (circle template; Fig. 1C) defined the required 90° relative phase (Kovacs et al. 2009a; McCulloch et al. 2021). The Lissajous plot also provides a target amplitude for performance (Buchanan 2004; Buchanan et al. 2007), in this case half-cycle 12 cm amplitudes for each hand (measured as combined radius of the template). Relative phase accuracy during training was not influenced by the LARC and RALC montages. This is consistent with the Vancleef et al. (2016) study that used a left M1 anode stimulation and produced no effect on relative phase accuracy, the current finding extends this to the right M1 anode case for this performance measure. Novel findings were revealed in the interaction found in the $\:{\varphi\:}_{SD}$ data set. This interaction revealed two main findings: 1) coordination was more variable (less stable) early in training in the RALC condition compared to the LARC and sham conditions within block 3; and 2) coordination variability decreased (stability increases) uniquely for each montage, leveling off at different points in training, LARC leveled off at B2, RALC leveled off at B4, and sham leveled off at B3 (Fig. 3B). Thus, a hemispheric asymmetry (medium sized effect *η*^*2*^_*p*_ = 0.09) based on a key performance measure associated with rhythmic bimanual tasks emerged early in training. Overall, performance accuracy and coordination variability of the 90° pattern were similar at the end of training for the three montages.

Research has shown that the left dominant hemisphere (in right-handed participants) primarily controls rhythmic bimanual movements such as in-phase and anti-phase, with C4 to C3 EEG coherence weaker than C3 to C4 EEG coherence (Serrien et al. 2006). This result was interpreted as a suppression of the non-dominant right hemisphere’s activity by the dominant left-hemisphere during rhythmic tasks. Research has demonstrated that cathode stimulation of left M1 produces inhibition, while anode stimulation increases excitability (Nitsche and Paulus 2000b, 2001). In the RALC condition, the anode may have increased right-hemisphere M1 (C4) excitability, while the cathode over left M1 (C3) may have produced inhibition. Thus, it is hypothesized that the RALC condition created a competition between the natural suppression by the left-hemisphere (Serrien et al. 2006) and the increased excitability in right M1 from the anode, thereby leading to larger variability early in training with the 90° pattern compared to the LARC condition. The use of the RALC montage did produce a benefit over sham stimulation in a non-dominant left-arm sequencing task, and it was hypothesized that the cathode over the left dominant hemisphere’s M1 dampened inhibitory input into the right-hemisphere and enhanced excitatory effects associated with the anode over the right hemisphere’s M1 (Vines et al. 2008a). The current findings do not support this hypothesis applied to bimanual tasks, and instead favor the idea that a competition emerged between inhibitory projections and excitability of right M1 because the two hemispheres had to work together (Serrien et al. 2003). The above hypotheses could be tested with cTBS during and after training to inhibit cortical activity to look for an influence on coordination stability.

The LARC montage resulted in larger radii (AMP) of the combined bimanual trace in the Lissajous plot than the sham montage. In the individual arm abduction-adduction amplitudes (see supplementary data S2), the LARC and RALC montages were associated with larger abduction-adduction motions compared to sham. These finding are interesting because they are consistent with recent findings demonstrating that bilateral stimulation of left and right M1 influence movement force in a positive performance direction. The study by Hikosaka and Aramaki (2021) used the same LARC and RALC montages as used here and found higher hand grip force in a bimanual task (both hands) associated with the LARC Montage compared to sham, with no difference between an LARC and RALC montage or RALC and sham. Lee and Kang (2023) reported that an LARC montage resulted in decreased force variability and increased coordination in a bimanual task compared to sham. Linking M1 to movement amplitude is consistent with recent fMRI findings suggesting that M1 activity is related to the execution of finger presses (Yokoi et al. 2018; Park et al. 2020). A bimanual learning study demonstrated increased peak-to-peak amplitude associated with single pulse MEPs from the FDI muscle (TMS of left M1 hand area, C3) after training with a bimanual 90° relative phase pattern with Lissajous feedback, with no correlation between performance accuracy (relative phase measures) and the increased peak-to-peak MEP amplitudes (Park et al. 2020). This suggests that M1 activity and increased excitability may be more related to the execution of component amplitudes than spatiotemporal performance, at least in the current training context with the Lissajous. In the bimanual force control work and the current study, concurrent augmented feedback in the form of visually matching a unified trace to a target was employed during training. This raises the issue of whether a general increase in cortical excitability under the anode electrode (M1) was responsible for the force and amplitude effects, or whether the excitability facilitated the matching between the action outcome and visual feedback. Another issue is the potential role of task instructions on the differences of spatiotemporal performance for participants in the sham group. It is our interpretation that the amplitude effects are not linked to the instructions. The instructions were not stated in terms of the performance variables, spatiotemporal or amplitude, only in terms of moving the dot along the curve (discovery learning). The lack of findings in the spatiotemporal data support the idea that the smaller amplitude in the sham and RALC group did not occur because the instructions biased amplitude performance more than spatiotemporal performance. Overall, larger bimanual amplitudes were associated with the LARC montage. It should be noted here that larger amplitude should not necessarily be interpreted as implying better performance. An analysis of the absolute error in amplitude did not reveal a significant difference between montages in training or retention.

The spatiotemporal data only measure required coordination, and not the specific scaling features of the task linked to amplitude. There is a separation between these two features of the bimanual action in that different limb amplitudes can be mapped to the same relative phase pattern (Ryu and Buchanan 2004; Buchanan and Ryu 2005). Work with single limb learning and transfer tasks, demonstrated that 90° relative phase is represented in a more abstract manner in that it can transfer across limb positions within the same limb, supine vs. prone, and across limbs from left to right and vice versa more readily than specific limb amplitudes (Buchanan et al. 2007). Moreover, a variety of movement amplitudes can be mapped to in-phase and anti-phase without influencing the accuracy or stability of performance (Buchanan and Ryu 2012). A study that stimulated left and right M1 in different conditions, revealed that anode stimulation of left M1 improved both contralateral and ipsilateral limb performance of a sequencing task, whereas anode stimulation of right M1 only benefited contralateral limb performance (Vines et al. 2008b). The LARC montage facilitated amplitude performance, this finding is consistent with the idea of the dominate hemisphere playing a larger role in bimanual coordination in general (Serrien et al. 2003) and also having a larger influence on the control of the two hands when used independently (Vines et al. 2008b; Schambra et al. 2011). A unique training finding that needs further study is that the RALC montage overall produced a larger abduction-adduction amplitude compared to sham, but not a larger bimanual amplitude based on the radius measure (statistically). Thus, tDCS anodal stimulation of left or right M1 during training produced an advantage associated with the task’s movement amplitude goal. This shows that control and coordination of the two hands together does not simply reduce to the understanding of the control of each single limb by itself (Swinnen 2002; Buchanan and Ryu 2006; Serrien et al. 2006; Kim et al. 2007, 2011).

In the present study, movement frequency was self-determined. Overall, movement frequency increased during training in all groups. This overall increase in movement frequency with Lissajous training is not a new finding in bimanual tasks without stimulation (Kovacs et al. 2009a; Buchanan and Wang 2012; McCulloch et al. 2021), and a specific montage in the current study did not alter that general trend. Increases in movement time (speed), reaction time, keystrokes, speed-accuracy assessments have been observed in unilateral tasks with tDCS applied to M1 (Vines et al. 2008a, b; Reis et al. 2009; Kim et al. 2021b), and recently in bimanual serial tasks with an anode over left or right M1 (Gomes-Osman and Field-Fote 2013) or only with an LARC montage (Furuya et al. 2014). Although the LARC montage was associated with the fastest frequencies, there were no statistical differences in frequency between montages during training.

### Retention performance and offline consolidation

After a 6-hr consolidation window, retention of bimanual performance was assessed with the use of concurrent feedback and the Lissajous plot. The analysis of the performance accuracy measure and coordination variability measure did not reveal any differences between stimulation conditions in the retention test. Thus, the asymmetry in coordination variability observed early in practice was not evident in retention. The analysis carried out on the delta values also did not reveal any differences between the end of training performance and retention test performance based on the relative phase data. This demonstrates off-line consolidation of the relative phase aspect of the task for both stimulation conditions and the sham condition. Consolidation over a time interval > 4 h. is a common finding in many unimanual tasks (Brashers-Krug et al. 1996; Robertson et al. 2004; Kim and Wright 2020; Kim et al. 2021a). A recent bimanual task using the same target relative phase and Lissajous training (without tDCS) demonstrated interference of the target pattern of 90° when a retention test followed an interference task, train on a 45° relative phase pattern, after only a 2-hr window (McCulloch et al. 2021). Thus, stimulation of M1, left or right-hemisphere, does not appear to have an influence on the consolidation of coordination accuracy and stability of bimanual relative phase patterns.

The retention amplitude data revealed a delayed emergence of a hemispheric asymmetry in amplitude. In training, bimanual amplitude (AMP) in LARC was larger than sham but not RALC. In retention after the 6-hr delay, LARC bimanual amplitude was found to be larger than both RALC and sham. Moreover, individual arm abduction-adduction amplitude in training was larger in LARC and RALC compared to sham, while after the 6-hr delay the retention test revealed abduction-adduction amplitude under LARC was larger than both RALC and sham (see suppl. data. S2). This demonstrates that the amplitude effects associated with LARC compared to sham were carried over into the retention test and extended to now include a difference compared to RALC for the bimanual measure of amplitude and the individual arm amplitudes. This finding suggests that the left-hemisphere plays a prominent role in generating overall movement amplitude for both arms in this task. This amplitude finding is consistent with findings showing an advantage of LARC stimulation in a unimanual sequential pinch force task (Schambra et al. 2011) and sequential typing task (King et al. 2020) for both left and right arms compared to RALC stimulation. Overall, right-arm amplitude was larger in both training and retention, with no interaction with montage. A recent bimanual force control study showed that an LARC montage was associated with larger force contributions from the right-arm compared to the left-arm compared to sham stimulation (Lee and Kang 2023); however, an RALC montage was not tested. The results reported in this study indicate that the difference in arm amplitude is mostly the result of the right-arm dominant participants, whereas the bimanual amplitude findings and combined arm adduction-abduction findings support a hemispheric asymmetry emerging across the 6-hr retention window.

## Limitations and conclusions

The current work revealed significant effects of tDCS montage on coordination variability and movement amplitude during training and retention of a bimanual action. The findings extend the work of Vancleef et al. (2016) by showing that investigation of bimanual coordination may be better served using bi-lateral M1 anode/cathode electrode placements and exploring the contribution of both hemispheres to such tasks, even in light of the work that shows the predominant role of the left-hemisphere in bimanual actions (Serrien et al. 2003). A limitation of the current study revolves around the use of the Lissajous for rapid training. The power of this form of augmented feedback may have reduced the impact of tDCS of M1 as related to rhythmic bimanual actions and relative phase measures. The Montage × Training interaction for the BW22 measure approached a standard level of accepted significance, while the Montage × Training interaction for variability was significant. These results should be interpreted with caution and suggest that future work exploring tDCS and learning of bimanual skills may need to utilize training protocols, e.g., blinking lights or auditory metronomes (Zanone and Kelso 1992; Summers et al. 1993), that do not afford such rapid training of the spatiotemporal aspect of the task to produce more robust effects from stimulation. The current study employed a single-blind protocol with only the participant blind to the stimulation, active or sham, a common protocol used with tDCS (Kim and Wright 2020; Lowenthal-Raz et al. 2024; Tseng et al. 2024; Yang et al. 2024). Double-blind protocols mask both participant and experimenter administering the test to the stimulation, active or sham, and is also used with tDCS (Giancatarina et al. 2024; Muller et al. 2024; Zhang et al. 2024). Some studies even use triple-blind protocols with the data analyst blind to the stimulation condition (Sato et al. 2024). Some may view the single-blind procedure as a weaker protocol in that the experimenter may inadvertently introduce bias into the testing context, a bias possibly reduced in the double-blind protocol. Recent meta-analysis reviews of tDCS studies rate the single-blind procedure as a medium risk for bias compared to double- or triple-blind protocols base on the Cochrane risk assessment tool (Liu et al. 2024; Usman et al. 2025). It’s important to note however, that the overall methodological quality of the study should be assessed on a variety of factors reported such as adverse effects of tDCS as well as blinding efficacy (Usman et al. 2025). In the current study, we employed a post-stimulation survey to test for tDCS blinding efficacy (Vancleef et al. 2016; Schneider et al. 2021), and found no statistical differences between montages to the effect that any group readily identified or experienced a noticeably different sensation (see supplemental information).

The most significant findings emerged in the bimanual amplitude data. The advantage associated with the LARC montage emerged in training compared to sham and was still present at the 6-hr retention interval in comparison to RALC and sham. It is our contention that this finding is consistent with the bimanual force outcomes found in recent studies that have used bi-lateral montages (Hikosaka and Aramaki 2021; Lee and Kang 2023) and left M1 anode montages (Jin et al. 2019a, b). The amplitude findings in this study and the force findings in other studies suggest that work going forward needs to focus on these variables with the stimulation of M1. As with the spatiotemporal variables, the Lissajous template creates a limitation in that it provided continuous feedback on the amplitude, however, the finding that the advantage of LARC training was also observed in retention overcomes this limitation to an extent. However, future work should again explore the use of tDCS on amplitude control and learning through different feedback techniques to explore why the LARC advantage emerged. It was expected that movement frequency would significantly increase with practice and remain at values for stable production of the trained 90° pattern. A limitation here is that a pacing signal was not used, thus there was a lot of individual variability in the frequency data. Research has shown that tDCS to SMA increases the stability of anti-phase bimanual coordination at faster movement frequencies (Carter et al. 2015, 2017). Overall, the results reveal that tDCS can play a role in revealing hemispheric contributions to the learning and consolidation of bimanual motor skills. It is our contention that more research comparing the specific influence of left (C3) and right (C4) M1 anode stimulation is warranted, especially regarding the control and learning of movement amplitude. Finally, it must be noted that the lack of focality of tDCS may inadvertently result in stimulation of motor areas beyond those targeted but possibly important for the performance of the bimanual task, such as the pre-motor region or SMA (Aramaki et al. 2006; Carter et al. 2015). Future work combining tDCS and TMS (more focal stimulation) is important to help clarify hemispheric specialization in bimanual tasks.

## Electronic supplementary material

Below is the link to the electronic supplementary material.

Supplementary Material 1

## Data Availability

The data that support the findings of this study are not openly available due to reasons of confidentiality/sensitivity but are available from the corresponding author upon reasonable request.
