# Supplementary material for: Application of bilateral tDCS over left and right M1 produces asymmetric training and retention effects when learning a rhythmic bimanual task
Source: Exp Brain Res. 2025 Mar 14;243(4):91. doi: 10.1007/s00221-025-07045-4 (PMC11909090; doi:10.1007/s00221-025-07045-4)
Supplement: Supplementary file 1 — Supplementary Material 1 [file 221_2025_7045_MOESM1_ESM.docx]

Supplementary data.

Section S1. Movement frequency by arm:

*Training.* The repeated measures ANOVA (procedure mixed SAS 9.4) performed on the left and right arm individual FRQ data did not reveal a significant effect of Arm, *F (1, 43) = .01, p = .91,* or Montage, *F (2, 28) = 0.45, p = .63.* The Montage Block (*F(12, 258) = 0.06, p = .99*), Montage Arm (*F(2, 43) = 0.07, p = .9*), Arm Block (*F(6, 258) = 0.06, p = .99*), and Montage Arm Block (*F(12, 258) = 0.05, p = 1.0*) interactions were non-significant*.* A significant main effect of Block was found, *F (6, 258) = 75.07, p < .0001,* $n_{p}^{2}=$ *.45)*. See main text for analysis of the averaged FRQ data for details on how movement frequency changed across blocks.

*Retention.* The 3 (Montage) 2 (Arm) ANOVA (procedure GLM) performed on the individual left and right arm FRQ data in the retention test did not find any significant effects: Montage (*F (2, 86) = 2.59, p = .08),* Arm, (*F (1, 86) = 0.00, p = .95),* Montage Arm, *(F (2, 86) = 0.01, p = .99)*.

The analysis of the frequency data indicates that the two arms were frequency locked throughout training and retention.

Section S2. Abduction-adduction amplitude by arm:

*Training.* The repeated measures ANOVA (procedure mixed SAS 9.4, post-hoc Tukey-Kramer, *p < .05*) of the individual abduction-adduction amplitudes of the arms revealed significant main effects of Montage, *F( 2, 28) = 9.05, p = .0009,* $n_{p}^{2}=$ *.39*, Arm, *F(1, 43) = 6.10, p = .0176,* $n_{p}^{2}=$ *.12,* and Block, *F(6, 258) = 30.07, p < .0001,* $n_{p}^{2}=$ *.41*. Post-hoc tests of the Montage effect found that arm amplitude during LARC (Mean = 11.6 cm, std. dev. = 1.9 cm) was larger (*t(28) = 4.09, p = .0009*) than sham (Mean = 9.9 cm, std. dev. 2.0 cm), RALC (Mean = 11.2 cm, std. dev. = 1.7 cm) was larger than sham *(t(28) = 3.23, p = .0087*), with no difference between LARC and RALC (*t(28) = 0.91, p = .64*). Overall, right-arm abduction-adduction amplitude (Mean = 11.17 cm, std. dev. = 2.07 cm) was larger than left-arm amplitude (Mean = 10.8 cm, std. dev. = 1.9 cm). Post-hoc tests of the Block effect found that arm amplitude was significantly smaller in B1 compared to all other blocks (all *t-value* reported have *p < .0001* unless reported): B1 < B2 (*t(258) = -9.71*), B1 < B3 (*t = -11.01*), B1 < B4 (*t = -12.15*), B1 < B5 (*t = -12.16*), B1 < B6 (*t = -12.04*), and B1 < B7 (*t = -11.94*). No other significant block effects were found. The Montage Block (*F(12, 258) = 1.34, p = .19*), Montage Arm (*F(2, 43) = 2.41, p = .09*), Arm Block (*F(6, 258) = 0.38, p = .89*), and Montage Arm Block (*F(12, 258) = 0.19, p = .99*) interactions were non-significant.

*Retention.* The 3 (Montage) 2 (Arm) ANOVA (procedure GLM) performed on the individual arm abduction-adduction amplitudes found a main effect of Montage, *F (2, 86) = 9.41, p = .0002,* $n_{p}^{2}=$ *.17.* Post hoc tests (Neuman-Keuls, *p < .05*) revealed that abduction-adduction arm amplitude in LARC (Mean = 11.8 cm, std. dev. = 1.04 cm) was significantly larger than RALC (Mean = 10.9 cm, std. dev. = 1.3 cm) and sham (Mean = 10.3 cm, std. dev. = 1.6 cm), with no difference between RALC and sham. The Arm effect (*F (1, 86) = 0.23, p = .63),* and Montage Arm interaction, *(F (2, 86) = 0.15, p = .86)*, were non-significant.

The analysis of the individual arm amplitudes revealed a main effect of arm only during training, with the right-arm producing small but significantly larger amplitudes than the left-arm. All participants were right-handed. Arm did not interact with Montage, suggesting the arm effect is most probably the result of the right-handed participants. The arm effect was not evident in the retention data. The Montage effect was still evident in the retention data, with the LARC montage associated with the largest arm amplitudes.

Section S3. Post-stimulation survey results:

A post tDCS survey was given to each participant. Table S1 displays the number of effects (often) associated with tDCS as reported by the participants in this experiment for each montage condition. All but one participant (sham montage) reported at least one affect.

Table S1.

|  | Itching | Pain | Burn | Warm | Pinch | Taste | Fatigue | Headache | Nausea | Other |
| --- | --- | --- | --- | --- | --- | --- | --- | --- | --- | --- |
| LARC | 16 | 5 | 8 | 9 | 1 | - | 4 | 2 | 1 | - |
| RALC | 16 | 4 | 9 | 7 | 4 | - | 5 | 1 | - | - |
| sham | 11 | 5 | 6 | 2 | 3 | 1 | 4 | 1 | 1 | - |

The survey also asked when the effect experienced started, how long stimulation lasted, and whether the participant experienced real stimulation, sham stimulation, or was unsure. The responses to the associated questions are reported in Table S2 by montage.

Table S2.

|  | First noticed affect | | Length of affect | | | Stimulation experienced | | |
| --- | --- | --- | --- | --- | --- | --- | --- | --- |
|  | Beginning | Middle | brief | minutes | Entire trial | Real | Sham | unsure |
| LARC | 14 | 2 | 3 | 9 | 3 | 11 | 1 | 4 |
| RALC | 12 | 4 | 2 | 7 | 6 | 9 | 4 | 3 |
| sham | 8 | 6 | 5 | 7 | 2 | 5 | 4 | 5 |

A Chi-Square test was performed on the stimulation experienced responses using the SAS (9.4) Procedure freq. The sham and unsure values were combined so as not to have less than 5 items in a cell for the Chi-Square test. The test returned a non-significant effect, *Χ(2) = 2.3102, p = 0.3150*.
